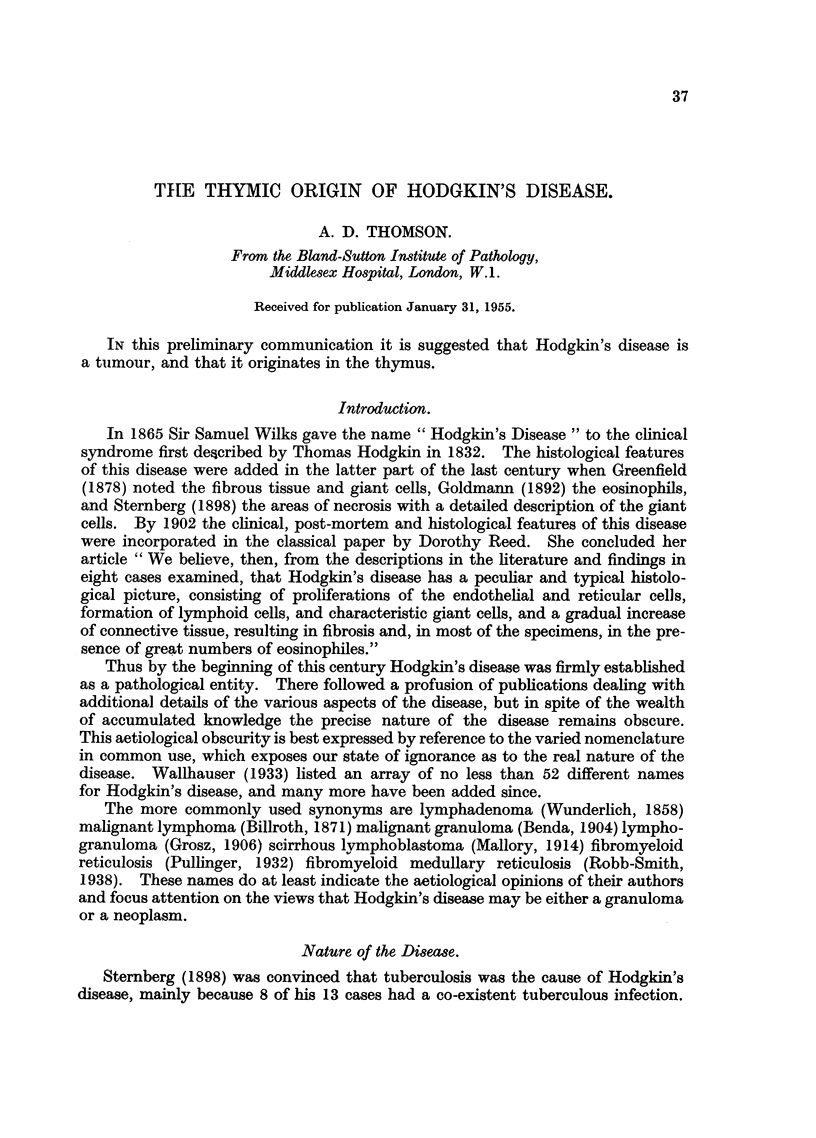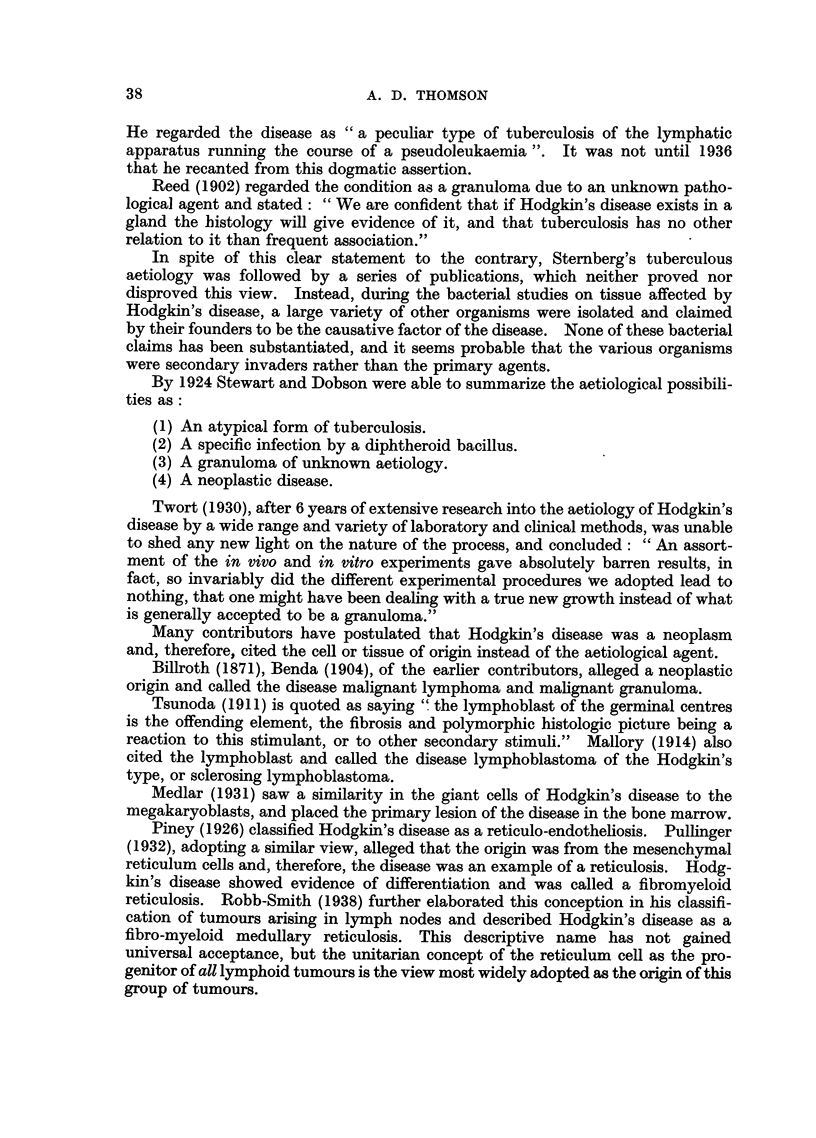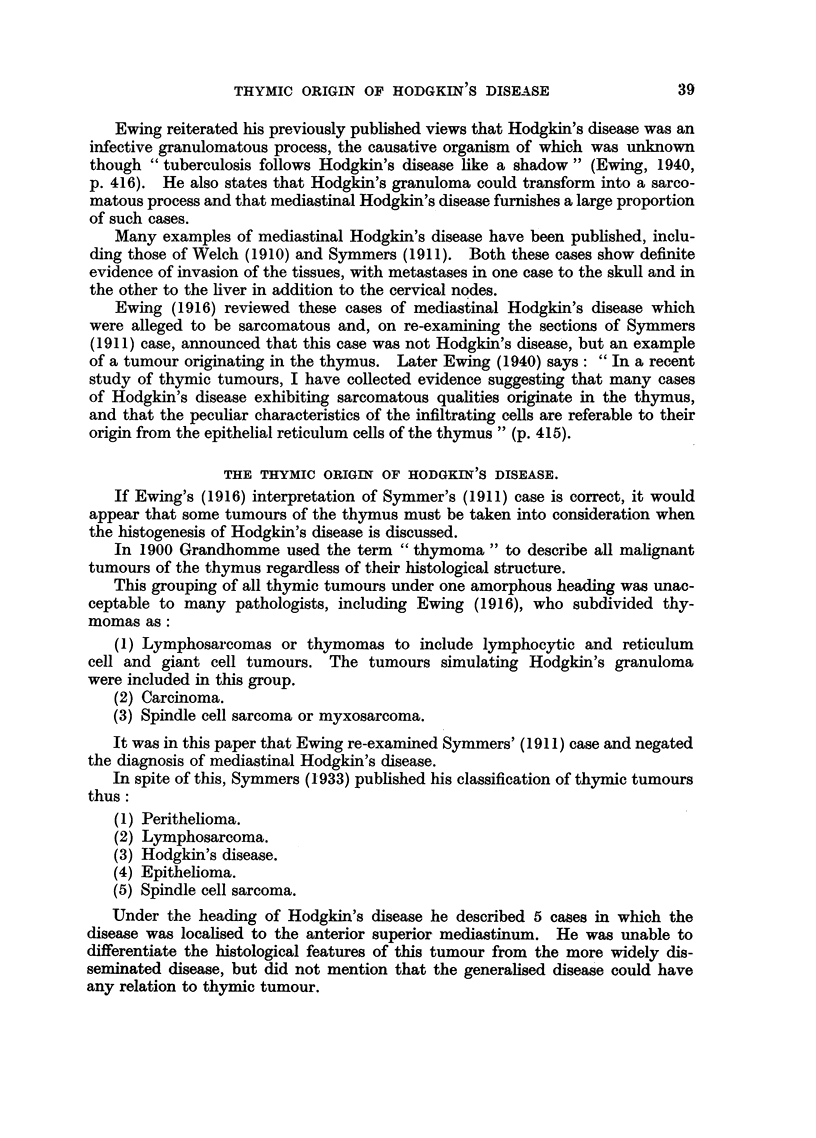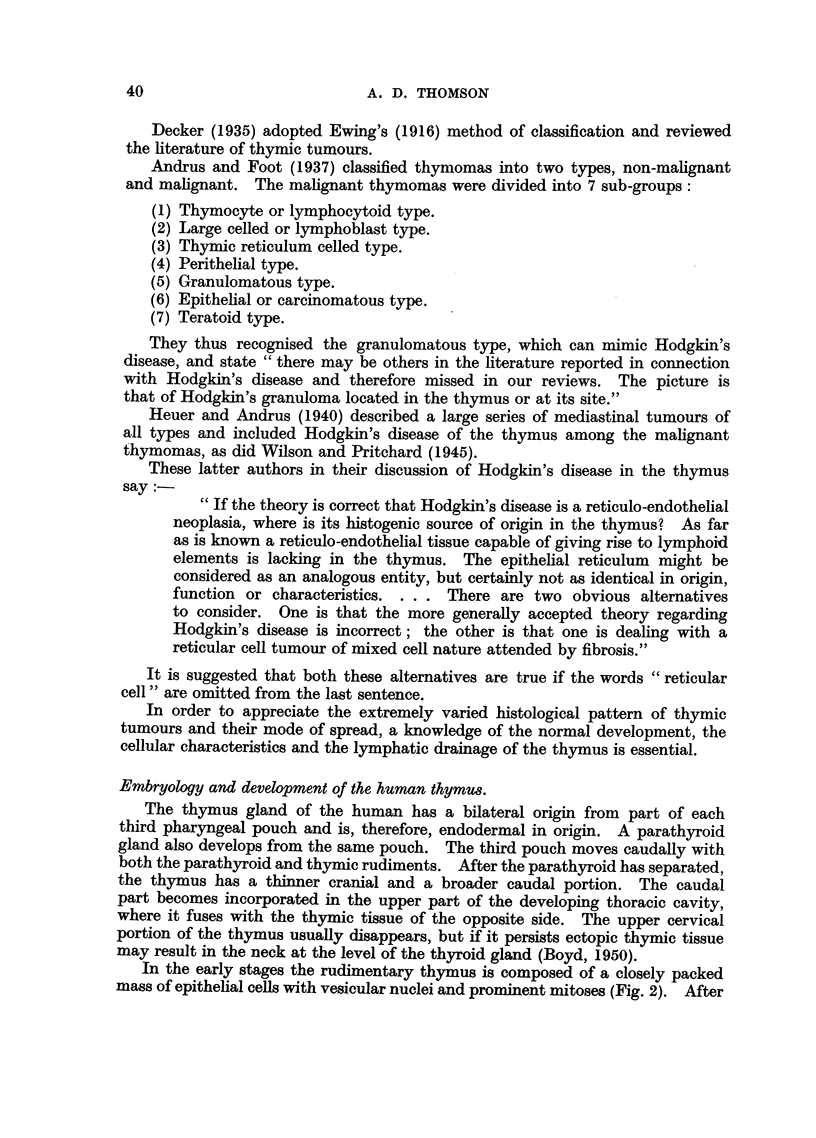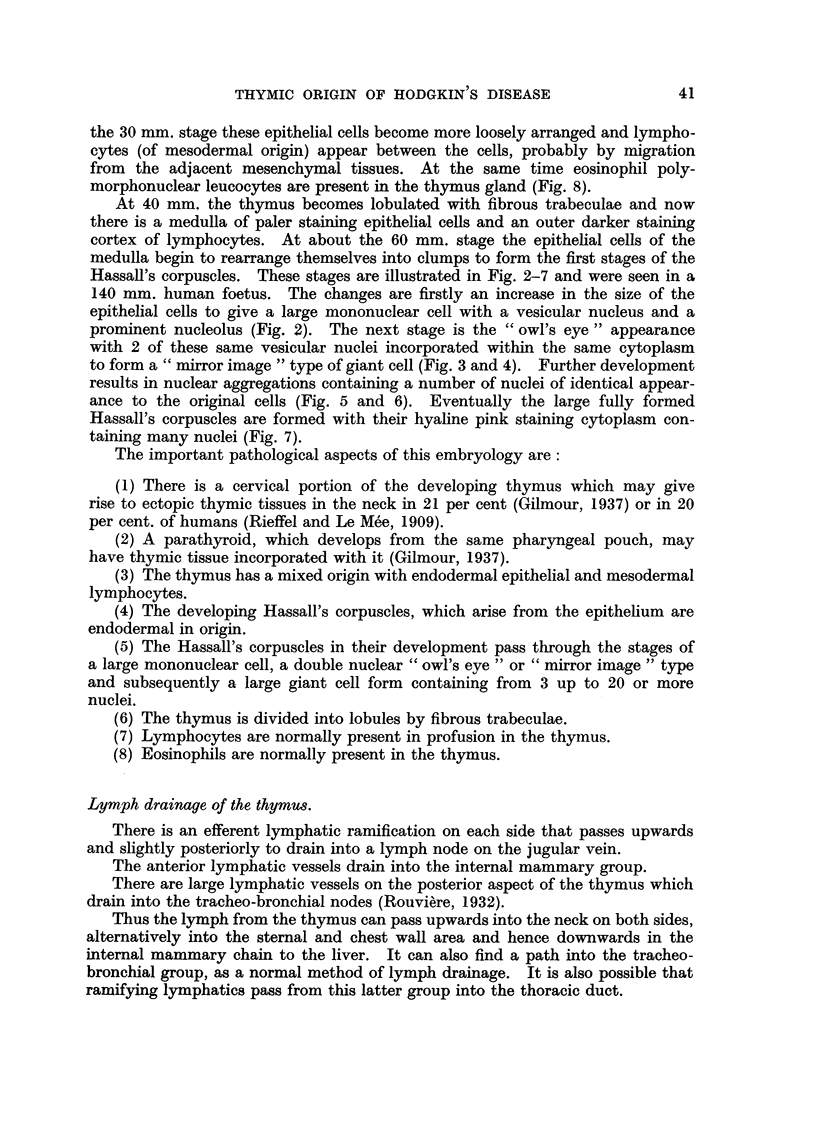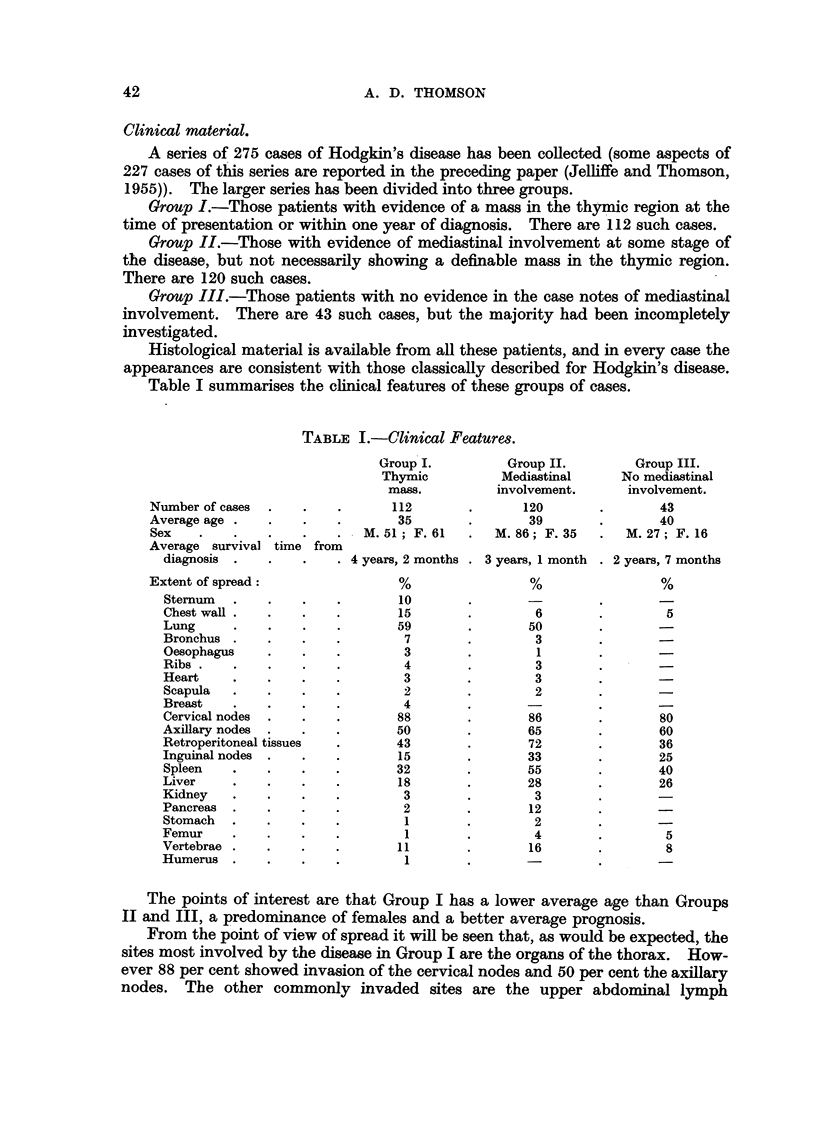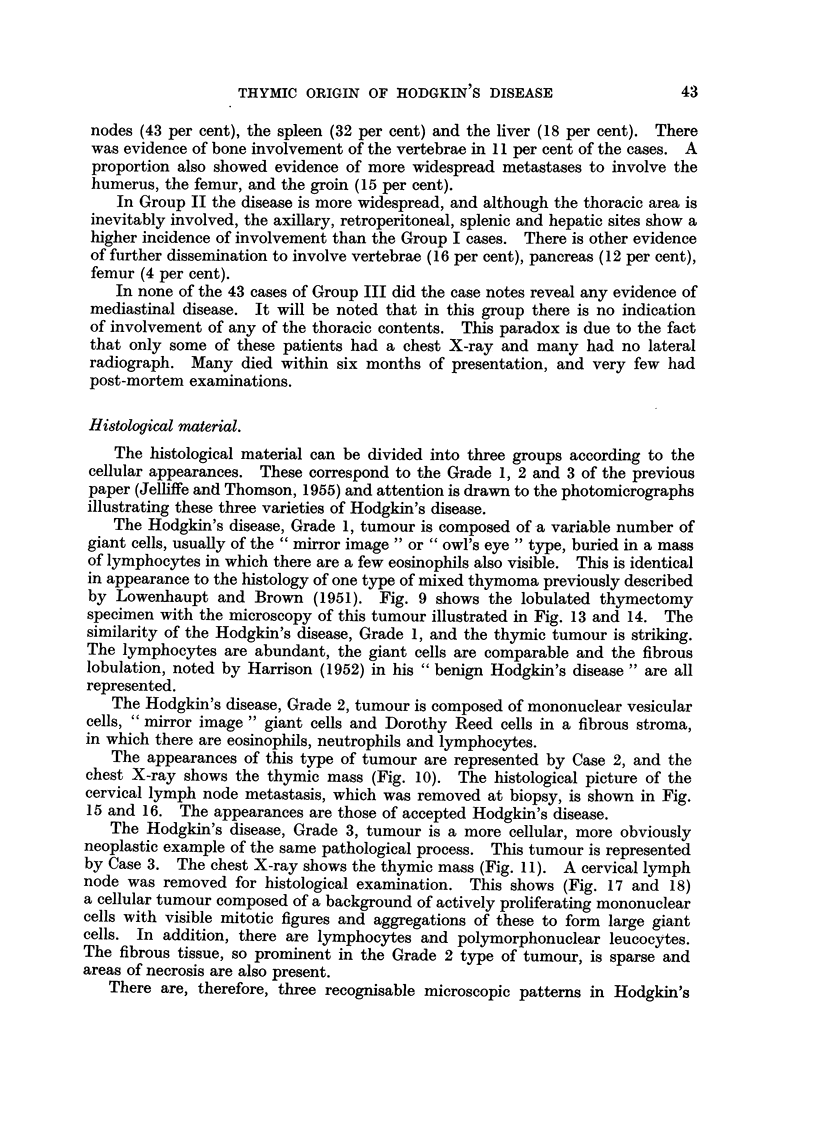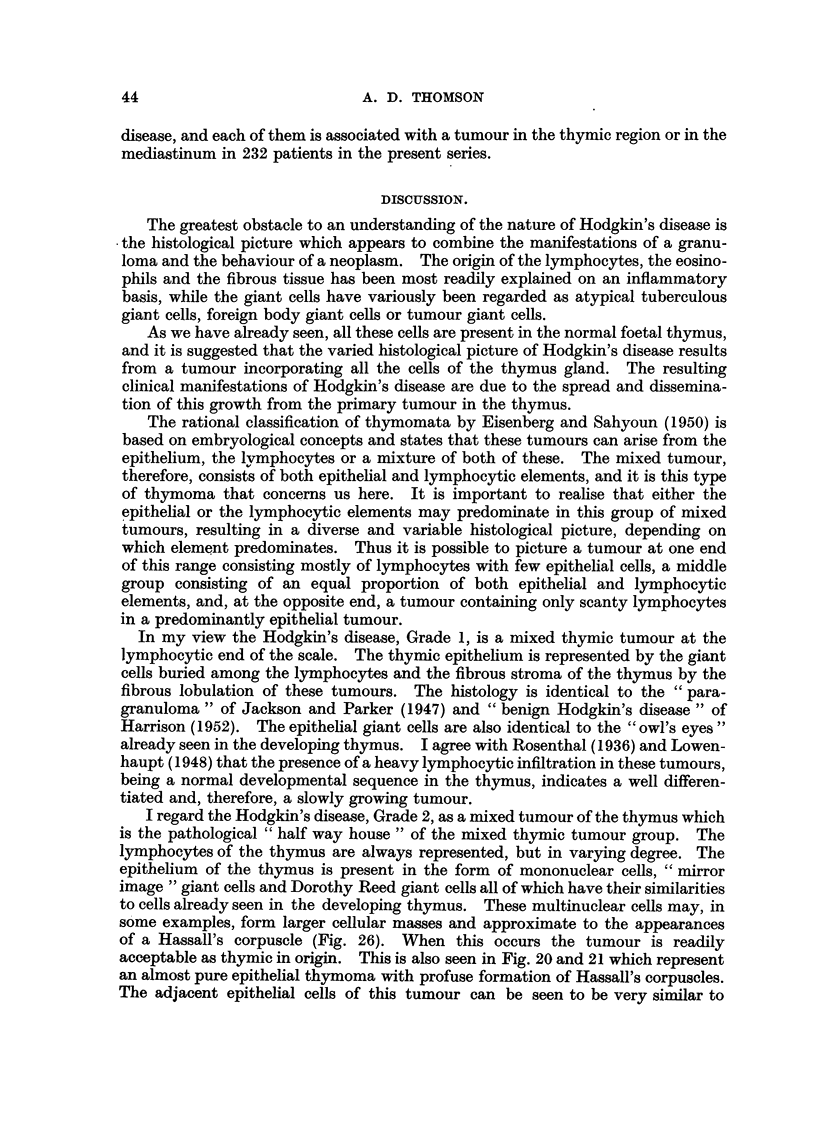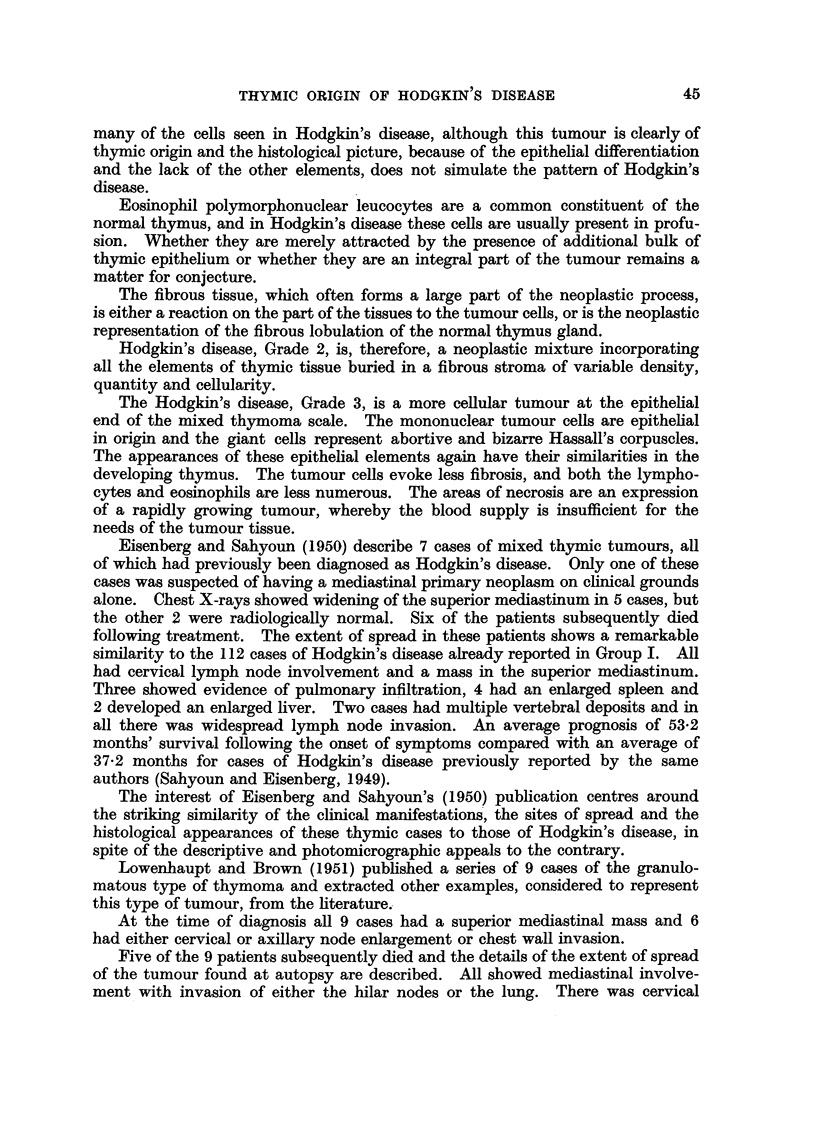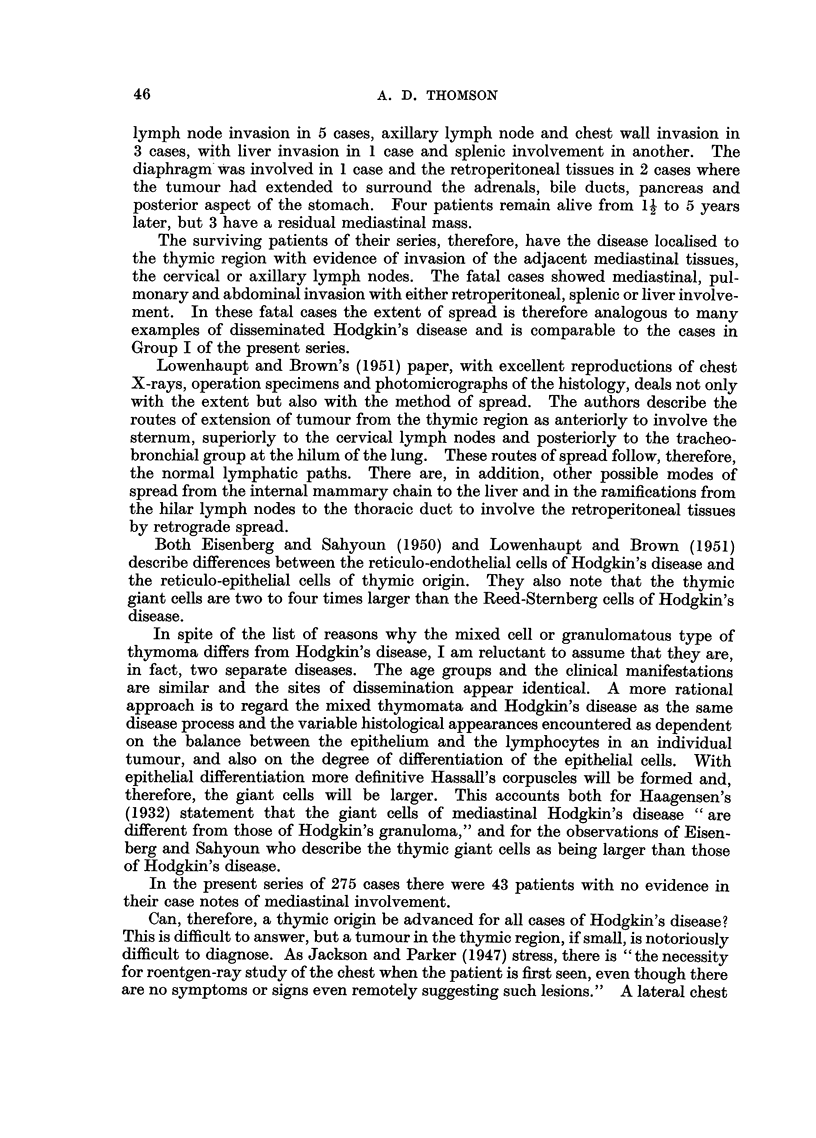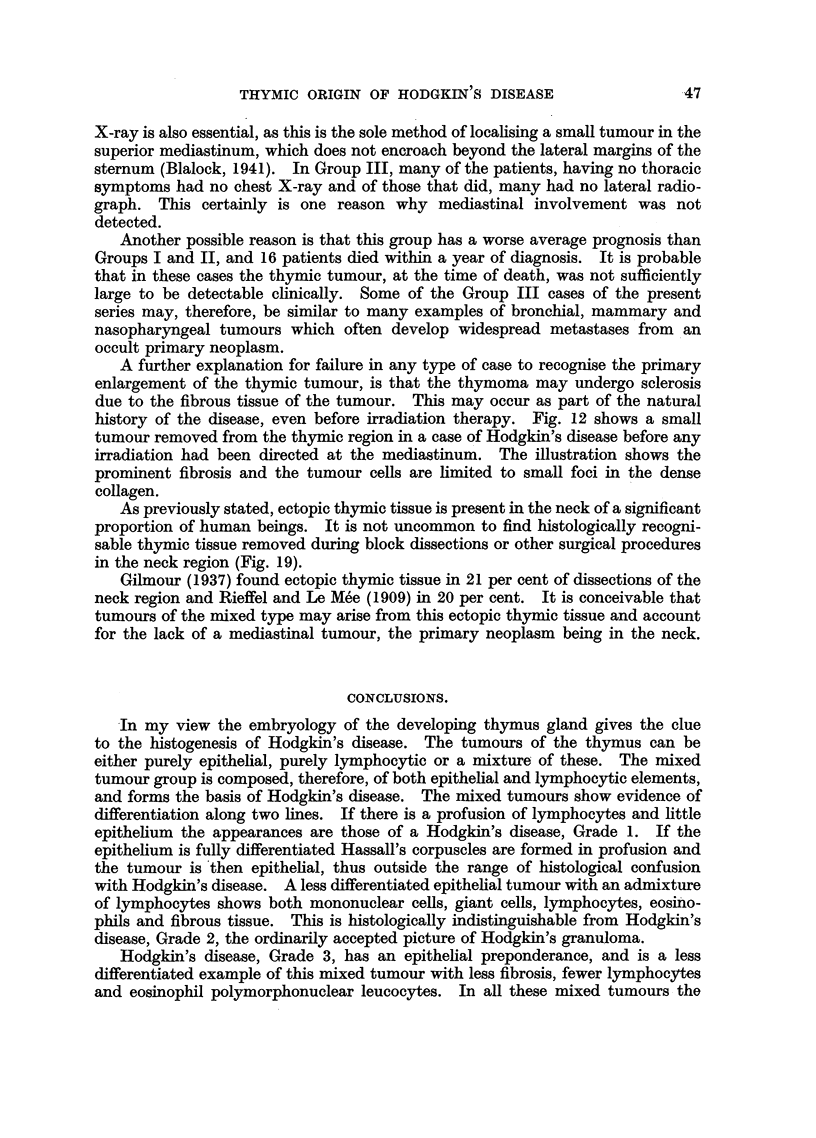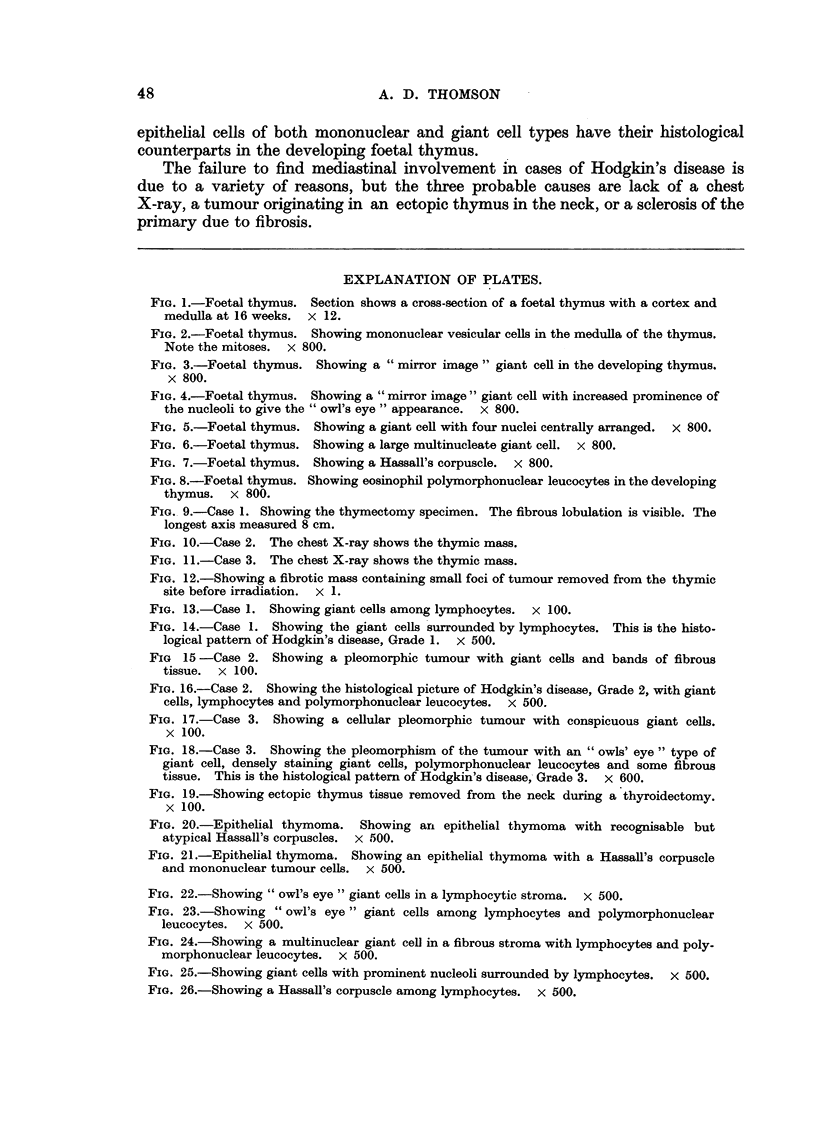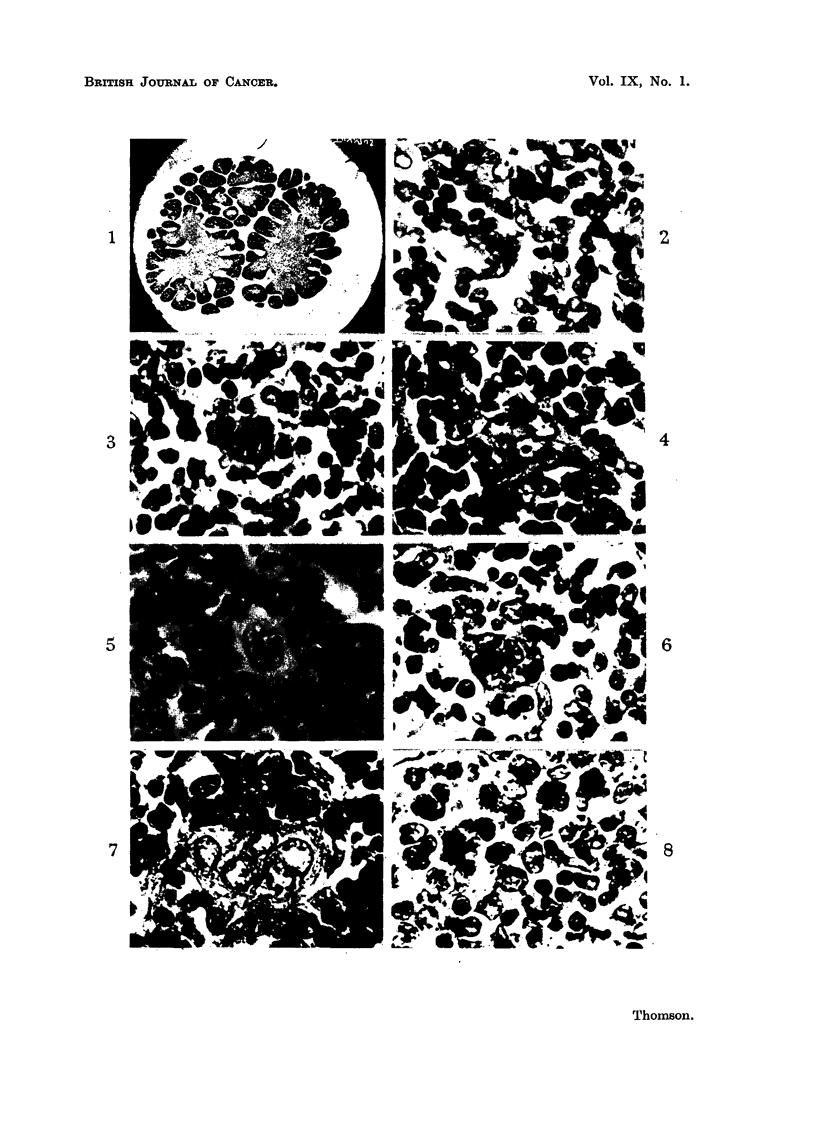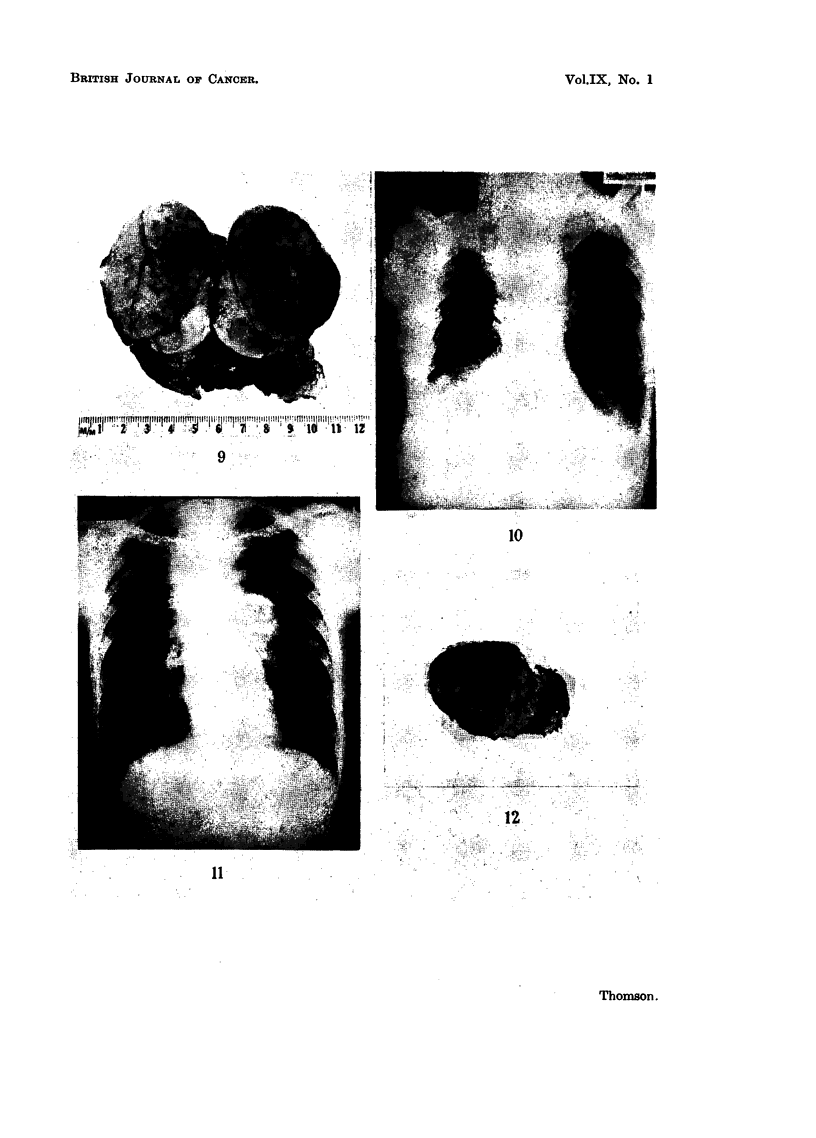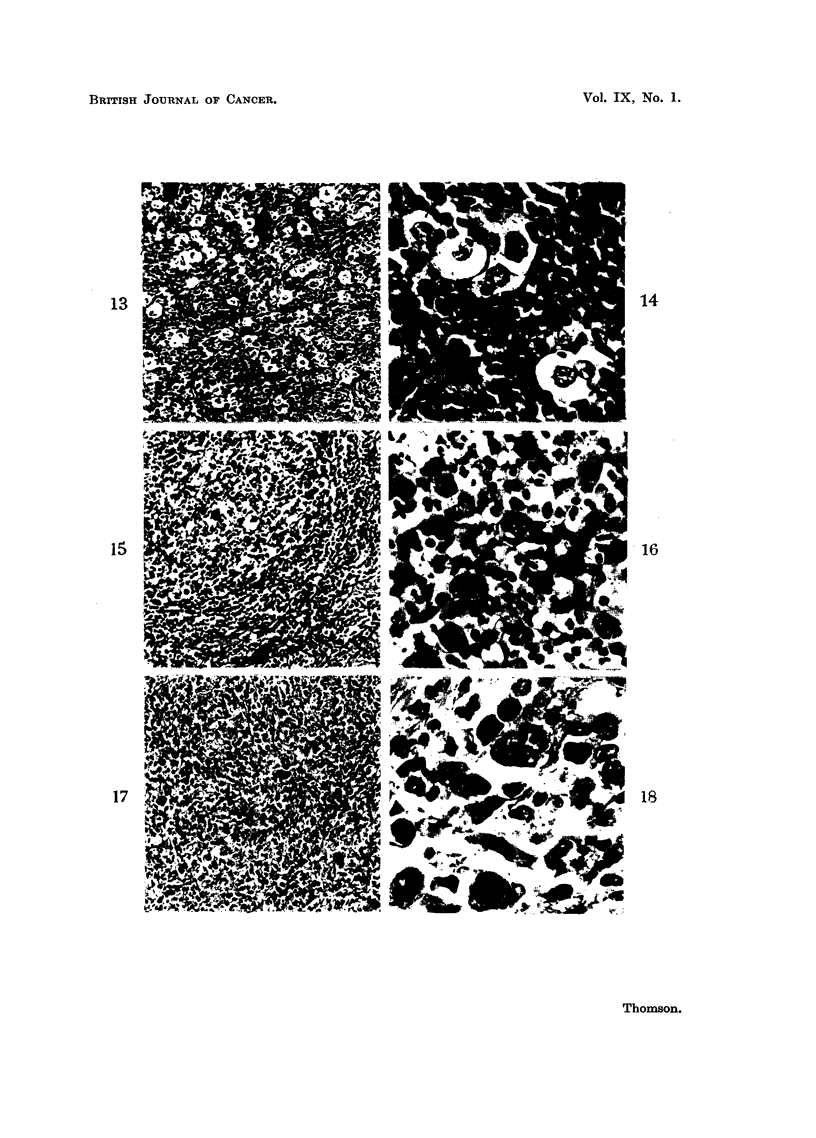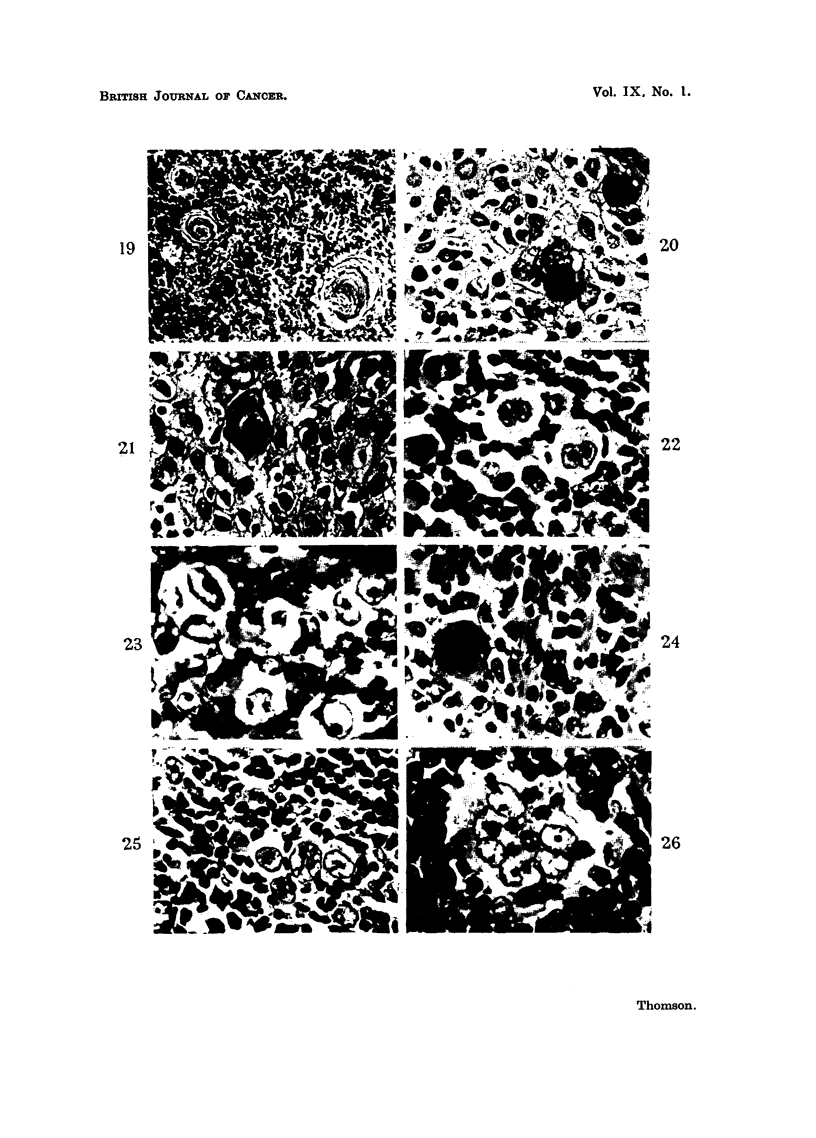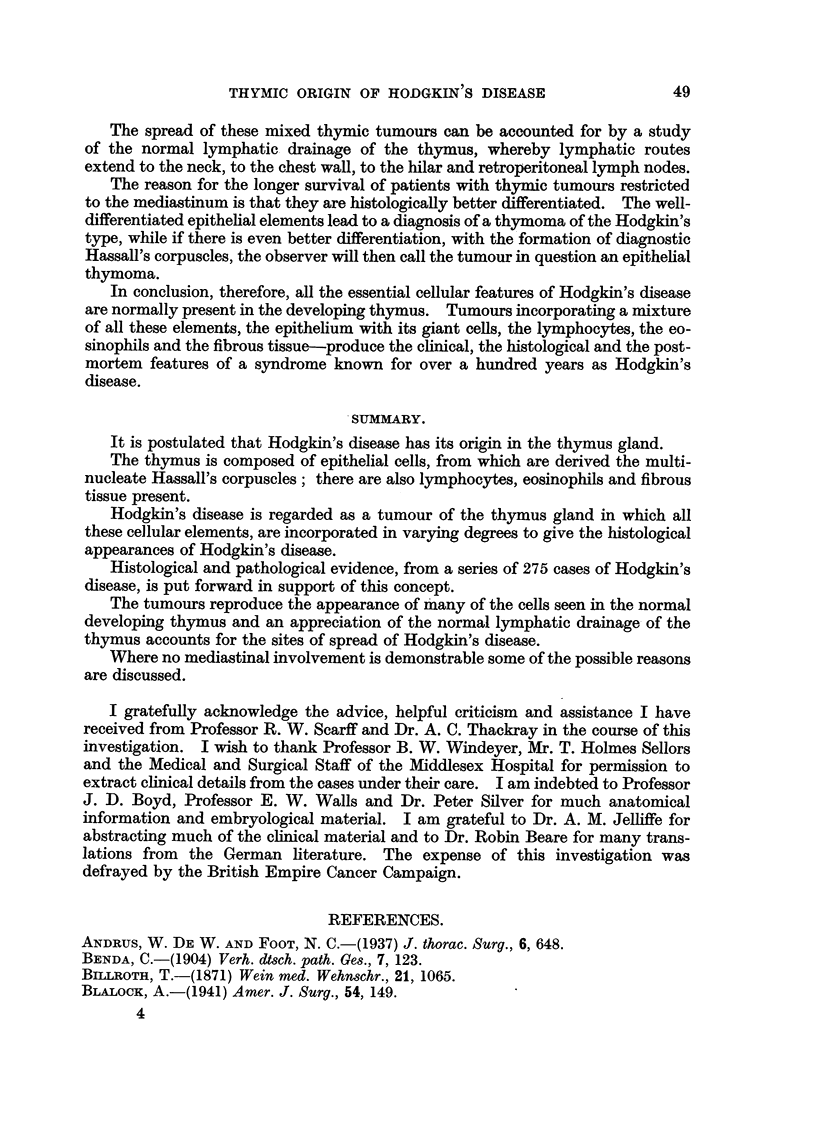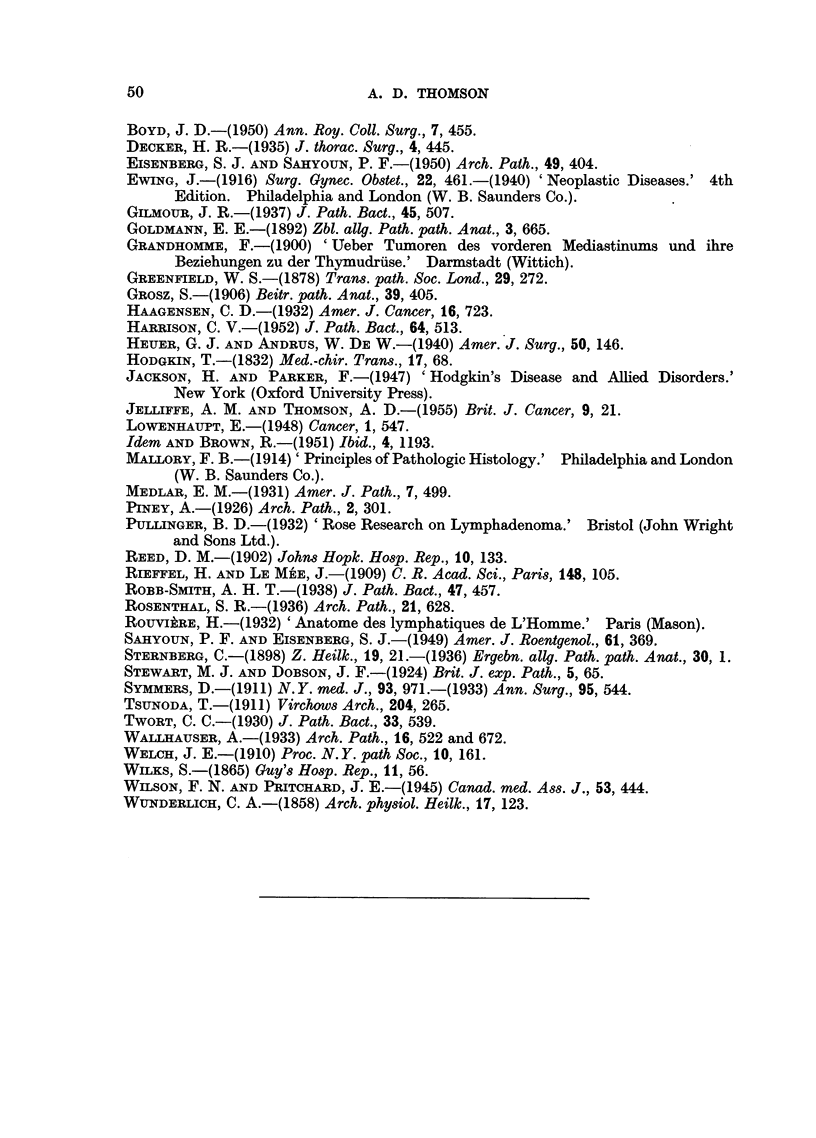# The Thymic Origin of Hodgkin's Disease

**DOI:** 10.1038/bjc.1955.4

**Published:** 1955-03

**Authors:** A. D. Thomson

## Abstract

**Images:**


### THE THYMIC ORIGIN OF HODGKIN'S DISEASE.

A. D. THOMSON.

*From the Bland-Sutton Institute of Pathology, Middlesex Hospital, London, W.1.*

Received for publication January 31, 1955.

IN this preliminary communication it is suggested that Hodgkin's disease is a tumour, and that it originates in the thymus.

### *Introduction.*

In 1865 Sir Samuel Wilks gave the name " Hodgkin's Disease " to the clinical syndrome first described by Thomas Hodgkin in 1832. The histological features of this disease were added in the latter part of the last century when Greenfield (1878) noted the fibrous tissue and giant cells, Goldmann (1892) the eosinophils, and Sternberg (1898) the areas of necrosis with a detailed description of the giant cells. By 1902 the clinical, post-mortem and histological features of this disease were incorporated in the classical paper by Dorothy Reed. She concluded her article " We believe, then, from the descriptions in the literature and findings in eight cases examined, that Hodgkin's disease has a peculiar and typical histological picture, consisting of proliferations of the endothelial and reticular cells, formation of lymphoid cells, and characteristic giant cells, and a gradual increase of connective tissue, resulting in fibrosis and, in most of the specimens, in the presence of great numbers of eosinophiles."

Thus by the beginning of this century Hodgkin's disease was firmly established as a pathological entity. There followed a profusion of publications dealing with additional details of the various aspects of the disease, but in spite of the wealth of accumulated knowledge the precise nature of the disease remains obscure. This aetiological obscurity is best expressed by reference to the varied nomenclature in common use, which exposes our state of ignorance as to the real nature of the disease. Wallhauser (1933) listed an array of no less than 52 different names for Hodgkin's disease, and many more have been added since.

The more commonly used synonyms are lymphadenoma (Wunderlich, 1858) malignant lymphoma (Billroth, 1871) malignant granuloma (Benda, 1904) lymphogranuloma (Grosz, 1906) scirrhous lymphoblastoma (Mallory, 1914) fibromyeloid reticulosis (Pullinger, 1932) fibromyeloid medullary reticulosis (Robb-Smith, 1938). These names do at least indicate the aetiological opinions of their authors and focus attention on the views that Hodgkin's disease may be either a granuloma or a neoplasm.

### *Nature of the Disease.*

Sternberg (1898) was convinced that tuberculosis was the cause of Hodgkin's disease, mainly because 8 of his 13 cases had a co-existent tuberculous infection.

He regarded the disease as " a peculiar type of tuberculosis of the lymphatic apparatus running the course of a pseudoleukaemia ". It was not until 1936 that he recanted from this dogmatic assertion.

Reed (1902) regarded the condition as a granuloma due to an unknown pathological agent and stated : " We are confident that if Hodgkin's disease exists in a gland the histology will give evidence of it, and that tuberculosis has no other relation to it than frequent association."

In spite of this clear statement to the contrary, Sternberg's tuberculous aetiology was followed by a series of publications, which neither proved nor disproved this view. Instead, during the bacterial studies on tissue affected by Hodgkin's disease, a large variety of other organisms were isolated and claimed by their founders to be the causative factor of the disease. None of these bacterial claims has been substantiated, and it seems probable that the various organisms were secondary invaders rather than the primary agents.

By 1924 Stewart and Dobson were able to summarize the aetiological possibilities as :

(1) An atypical form of tuberculosis.
(2) A specific infection by a diphtheroid bacillus.
(3) A granuloma of unknown aetiology.
(4) A neoplastic disease.

Twort (1930), after 6 years of extensive research into the aetiology of Hodgkin's disease by a wide range and variety of laboratory and clinical methods, was unable to shed any new light on the nature of the process, and concluded : " An assortment of the *in vivo* and *in vitro* experiments gave absolutely barren results, in fact, so invariably did the different experimental procedures we adopted lead to nothing, that one might have been dealing with a true new growth instead of what is generally accepted to be a granuloma."

Many contributors have postulated that Hodgkin's disease was a neoplasm and, therefore, cited the cell or tissue of origin instead of the aetiological agent.

Billroth (1871), Benda (1904), of the earlier contributors, alleged a neoplastic origin and called the disease malignant lymphoma and malignant granuloma.

Tsunoda (1911) is quoted as saying " the lymphoblast of the germinal centres is the offending element, the fibrosis and polymorphic histologic picture being a reaction to this stimulant, or to other secondary stimuli." Mallory (1914) also cited the lymphoblast and called the disease lymphoblastoma of the Hodgkin's type, or sclerosing lymphoblastoma.

Medlar (1931) saw a similarity in the giant cells of Hodgkin's disease to the megakaryoblasts, and placed the primary lesion of the disease in the bone marrow.

Piney (1926) classified Hodgkin's disease as a reticulo-endotheliosis. Pullinger (1932), adopting a similar view, alleged that the origin was from the mesenchymal reticulum cells and, therefore, the disease was an example of a reticulosis. Hodgkin's disease showed evidence of differentiation and was called a fibromyeloid reticulosis. Robb-Smith (1938) further elaborated this conception in his classification of tumours arising in lymph nodes and described Hodgkin's disease as a fibro-myeloid medullary reticulosis. This descriptive name has not gained universal acceptance, but the unitarian concept of the reticulum cell as the progenitor of *all* lymphoid tumours is the view most widely adopted as the origin of this group of tumours.

Ewing reiterated his previously published views that Hodgkin's disease was an infective granulomatous process, the causative organism of which was unknown though "tuberculosis follows Hodgkin's disease like a shadow" (Ewing, 1940, p. 416). He also states that Hodgkin's granuloma could transform into a sarcomatous process and that mediastinal Hodgkin's disease furnishes a large proportion of such cases.

Many examples of mediastinal Hodgkin's disease have been published, including those of Welch (1910) and Symmers (1911). Both these cases show definite evidence of invasion of the tissues, with metastases in one case to the skull and in the other to the liver in addition to the cervical nodes.

Ewing (1916) reviewed these cases of mediastinal Hodgkin's disease which were alleged to be sarcomatous and, on re-examining the sections of Symmers (1911) case, announced that this case was not Hodgkin's disease, but an example of a tumour originating in the thymus. Later Ewing (1940) says: "In a recent study of thymic tumours, I have collected evidence suggesting that many cases of Hodgkin's disease exhibiting sarcomatous qualities originate in the thymus, and that the peculiar characteristics of the infiltrating cells are referable to their origin from the epithelial reticulum cells of the thymus" (p. 415).

### THE THYMIC ORIGIN OF HODGKIN'S DISEASE.

If Ewing's (1916) interpretation of Symmer's (1911) case is correct, it would appear that some tumours of the thymus must be taken into consideration when the histogenesis of Hodgkin's disease is discussed.

In 1900 Grandhomme used the term "thymoma" to describe all malignant tumours of the thymus regardless of their histological structure.

This grouping of all thymic tumours under one amorphous heading was unacceptable to many pathologists, including Ewing (1916), who subdivided thymomas as:

(1) Lymphosarcomas or thymomas to include lymphocytic and reticulum cell and giant cell tumours. The tumours simulating Hodgkin's granuloma were included in this group.
(2) Carcinoma.
(3) Spindle cell sarcoma or myxosarcoma.

It was in this paper that Ewing re-examined Symmers' (1911) case and negated the diagnosis of mediastinal Hodgkin's disease.

In spite of this, Symmers (1933) published his classification of thymic tumours thus:

(1) Perithelioma.
(2) Lymphosarcoma.
(3) Hodgkin's disease.
(4) Epithelioma.
(5) Spindle cell sarcoma.

Under the heading of Hodgkin's disease he described 5 cases in which the disease was localised to the anterior superior mediastinum. He was unable to differentiate the histological features of this tumour from the more widely disseminated disease, but did not mention that the generalised disease could have any relation to thymic tumour.

Decker (1935) adopted Ewing's (1916) method of classification and reviewed the literature of thymic tumours.

Andrus and Foot (1937) classified thymomas into two types, non-malignant and malignant. The malignant thymomas were divided into 7 sub-groups :

(1) Thymocyte or lymphocytoid type.
(2) Large celled or lymphoblast type.
(3) Thymic reticulum celled type.
(4) Perithelial type.
(5) Granulomatous type.
(6) Epithelial or carcinomatous type.
(7) Teratoid type.

They thus recognised the granulomatous type, which can mimic Hodgkin's disease, and state " there may be others in the literature reported in connection with Hodgkin's disease and therefore missed in our reviews. The picture is that of Hodgkin's granuloma located in the thymus or at its site."

Heuer and Andrus (1940) described a large series of mediastinal tumours of all types and included Hodgkin's disease of the thymus among the malignant thymomas, as did Wilson and Pritchard (1945).

These latter authors in their discussion of Hodgkin's disease in the thymus say :—

> " If the theory is correct that Hodgkin's disease is a reticulo-endothelial neoplasia, where is its histogenic source of origin in the thymus? As far as is known a reticulo-endothelial tissue capable of giving rise to lymphoid elements is lacking in the thymus. The epithelial reticulum might be considered as an analogous entity, but certainly not as identical in origin, function or characteristics. . . . There are two obvious alternatives to consider. One is that the more generally accepted theory regarding Hodgkin's disease is incorrect ; the other is that one is dealing with a reticular cell tumour of mixed cell nature attended by fibrosis."

It is suggested that both these alternatives are true if the words " reticular cell " are omitted from the last sentence.

In order to appreciate the extremely varied histological pattern of thymic tumours and their mode of spread, a knowledge of the normal development, the cellular characteristics and the lymphatic drainage of the thymus is essential.

*Embryology and development of the human thymus.*

The thymus gland of the human has a bilateral origin from part of each third pharyngeal pouch and is, therefore, endodermal in origin. A parathyroid gland also develops from the same pouch. The third pouch moves caudally with both the parathyroid and thymic rudiments. After the parathyroid has separated, the thymus has a thinner cranial and a broader caudal portion. The caudal part becomes incorporated in the upper part of the developing thoracic cavity, where it fuses with the thymic tissue of the opposite side. The upper cervical portion of the thymus usually disappears, but if it persists ectopic thymic tissue may result in the neck at the level of the thyroid gland (Boyd, 1950).

In the early stages the rudimentary thymus is composed of a closely packed mass of epithelial cells with vesicular nuclei and prominent mitoses (Fig. 2). After the 30 mm. stage these epithelial cells become more loosely arranged and lymphocytes (of mesodermal origin) appear between the cells, probably by migration from the adjacent mesenchymal tissues. At the same time eosinophil polymorphonuclear leucocytes are present in the thymus gland (Fig. 8).

At 40 mm. the thymus becomes lobulated with fibrous trabeculae and now there is a medulla of paler staining epithelial cells and an outer darker staining cortex of lymphocytes. At about the 60 mm. stage the epithelial cells of the medulla begin to rearrange themselves into clumps to form the first stages of the Hassall's corpuscles. These stages are illustrated in Fig. 2–7 and were seen in a 140 mm. human foetus. The changes are firstly an increase in the size of the epithelial cells to give a large mononuclear cell with a vesicular nucleus and a prominent nucleolus (Fig. 2). The next stage is the " owl's eye " appearance with 2 of these same vesicular nuclei incorporated within the same cytoplasm to form a " mirror image " type of giant cell (Fig. 3 and 4). Further development results in nuclear aggregations containing a number of nuclei of identical appearance to the original cells (Fig. 5 and 6). Eventually the large fully formed Hassall's corpuscles are formed with their hyaline pink staining cytoplasm containing many nuclei (Fig. 7).

The important pathological aspects of this embryology are :

(1) There is a cervical portion of the developing thymus which may give rise to ectopic thymic tissues in the neck in 21 per cent (Gilmour, 1937) or in 20 per cent. of humans (Rieffel and Le Mée, 1909).

(2) A parathyroid, which develops from the same pharyngeal pouch, may have thymic tissue incorporated with it (Gilmour, 1937).

(3) The thymus has a mixed origin with endodermal epithelial and mesodermal lymphocytes.

(4) The developing Hassall's corpuscles, which arise from the epithelium are endodermal in origin.

(5) The Hassall's corpuscles in their development pass through the stages of a large mononuclear cell, a double nuclear " owl's eye " or " mirror image " type and subsequently a large giant cell form containing from 3 up to 20 or more nuclei.

(6) The thymus is divided into lobules by fibrous trabeculae.

(7) Lymphocytes are normally present in profusion in the thymus.

(8) Eosinophils are normally present in the thymus.

*Lymph drainage of the thymus.*

There is an efferent lymphatic ramification on each side that passes upwards and slightly posteriorly to drain into a lymph node on the jugular vein.

The anterior lymphatic vessels drain into the internal mammary group.

There are large lymphatic vessels on the posterior aspect of the thymus which drain into the tracheo-bronchial nodes (Rouvière, 1932).

Thus the lymph from the thymus can pass upwards into the neck on both sides, alternatively into the sternal and chest wall area and hence downwards in the internal mammary chain to the liver. It can also find a path into the tracheo-bronchial group, as a normal method of lymph drainage. It is also possible that ramifying lymphatics pass from this latter group into the thoracic duct.

*Clinical material.*

A series of 275 cases of Hodgkin's disease has been collected (some aspects of 227 cases of this series are reported in the preceding paper (Jelliffe and Thomson, 1955)). The larger series has been divided into three groups.

*Group I.*—Those patients with evidence of a mass in the thymic region at the time of presentation or within one year of diagnosis. There are 112 such cases.

*Group II.*—Those with evidence of mediastinal involvement at some stage of the disease, but not necessarily showing a definable mass in the thymic region. There are 120 such cases.

*Group III.*—Those patients with no evidence in the case notes of mediastinal involvement. There are 43 such cases, but the majority had been incompletely investigated.

Histological material is available from all these patients, and in every case the appearances are consistent with those classically described for Hodgkin's disease.

Table I summarises the clinical features of these groups of cases.

TABLE I.—*Clinical Features.*

| | Group I. Thymic mass. | Group II. Mediastinal involvement. | Group III. No mediastinal involvement. |
|---|---|---|---|
| Number of cases | 112 | 120 | 43 |
| Average age | 35 | 39 | 40 |
| Sex | M. 51 ; F. 61 | M. 86 ; F. 35 | M. 27 ; F. 16 |
| Average survival time from diagnosis | 4 years, 2 months | 3 years, 1 month | 2 years, 7 months |
| Extent of spread : | % | % | % |
| Sternum | 10 | — | — |
| Chest wall | 15 | 6 | 5 |
| Lung | 59 | 50 | — |
| Bronchus | 7 | 3 | — |
| Oesophagus | 3 | 1 | — |
| Ribs | 4 | 3 | — |
| Heart | 3 | 3 | — |
| Scapula | 2 | 2 | — |
| Breast | 4 | — | — |
| Cervical nodes | 88 | 86 | 80 |
| Axillary nodes | 50 | 65 | 60 |
| Retroperitoneal tissues | 43 | 72 | 36 |
| Inguinal nodes | 15 | 33 | 25 |
| Spleen | 32 | 55 | 40 |
| Liver | 18 | 28 | 26 |
| Kidney | 3 | 3 | — |
| Pancreas | 2 | 12 | — |
| Stomach | 1 | 2 | — |
| Femur | 1 | 4 | 5 |
| Vertebrae | 11 | 16 | 8 |
| Humerus | 1 | — | — |

The points of interest are that Group I has a lower average age than Groups II and III, a predominance of females and a better average prognosis.

From the point of view of spread it will be seen that, as would be expected, the sites most involved by the disease in Group I are the organs of the thorax. However 88 per cent showed invasion of the cervical nodes and 50 per cent the axillary nodes. The other commonly invaded sites are the upper abdominal lymph nodes (43 per cent), the spleen (32 per cent) and the liver (18 per cent). There was evidence of bone involvement of the vertebrae in 11 per cent of the cases. A proportion also showed evidence of more widespread metastases to involve the humerus, the femur, and the groin (15 per cent).

In Group II the disease is more widespread, and although the thoracic area is inevitably involved, the axillary, retroperitoneal, splenic and hepatic sites show a higher incidence of involvement than the Group I cases. There is other evidence of further dissemination to involve vertebrae (16 per cent), pancreas (12 per cent), femur (4 per cent).

In none of the 43 cases of Group III did the case notes reveal any evidence of mediastinal disease. It will be noted that in this group there is no indication of involvement of any of the thoracic contents. This paradox is due to the fact that only some of these patients had a chest X-ray and many had no lateral radiograph. Many died within six months of presentation, and very few had post-mortem examinations.

*Histological material.*

The histological material can be divided into three groups according to the cellular appearances. These correspond to the Grade 1, 2 and 3 of the previous paper (Jelliffe and Thomson, 1955) and attention is drawn to the photomicrographs illustrating these three varieties of Hodgkin's disease.

The Hodgkin's disease, Grade 1, tumour is composed of a variable number of giant cells, usually of the " mirror image " or " owl's eye " type, buried in a mass of lymphocytes in which there are a few eosinophils also visible. This is identical in appearance to the histology of one type of mixed thymoma previously described by Lowenhaupt and Brown (1951). Fig. 9 shows the lobulated thymectomy specimen with the microscopy of this tumour illustrated in Fig. 13 and 14. The similarity of the Hodgkin's disease, Grade 1, and the thymic tumour is striking. The lymphocytes are abundant, the giant cells are comparable and the fibrous lobulation, noted by Harrison (1952) in his " benign Hodgkin's disease " are all represented.

The Hodgkin's disease, Grade 2, tumour is composed of mononuclear vesicular cells, " mirror image " giant cells and Dorothy Reed cells in a fibrous stroma, in which there are eosinophils, neutrophils and lymphocytes.

The appearances of this type of tumour are represented by Case 2, and the chest X-ray shows the thymic mass (Fig. 10). The histological picture of the cervical lymph node metastasis, which was removed at biopsy, is shown in Fig. 15 and 16. The appearances are those of accepted Hodgkin's disease.

The Hodgkin's disease, Grade 3, tumour is a more cellular, more obviously neoplastic example of the same pathological process. This tumour is represented by Case 3. The chest X-ray shows the thymic mass (Fig. 11). A cervical lymph node was removed for histological examination. This shows (Fig. 17 and 18) a cellular tumour composed of a background of actively proliferating mononuclear cells with visible mitotic figures and aggregations of these to form large giant cells. In addition, there are lymphocytes and polymorphonuclear leucocytes. The fibrous tissue, so prominent in the Grade 2 type of tumour, is sparse and areas of necrosis are also present.

There are, therefore, three recognisable microscopic patterns in Hodgkin's disease, and each of them is associated with a tumour in the thymic region or in the mediastinum in 232 patients in the present series.

### DISCUSSION.

The greatest obstacle to an understanding of the nature of Hodgkin's disease is the histological picture which appears to combine the manifestations of a granuloma and the behaviour of a neoplasm. The origin of the lymphocytes, the eosinophils and the fibrous tissue has been most readily explained on an inflammatory basis, while the giant cells have variously been regarded as atypical tuberculous giant cells, foreign body giant cells or tumour giant cells.

As we have already seen, all these cells are present in the normal foetal thymus, and it is suggested that the varied histological picture of Hodgkin's disease results from a tumour incorporating all the cells of the thymus gland. The resulting clinical manifestations of Hodgkin's disease are due to the spread and dissemination of this growth from the primary tumour in the thymus.

The rational classification of thymomata by Eisenberg and Sahyoun (1950) is based on embryological concepts and states that these tumours can arise from the epithelium, the lymphocytes or a mixture of both of these. The mixed tumour, therefore, consists of both epithelial and lymphocytic elements, and it is this type of thymoma that concerns us here. It is important to realise that either the epithelial or the lymphocytic elements may predominate in this group of mixed tumours, resulting in a diverse and variable histological picture, depending on which element predominates. Thus it is possible to picture a tumour at one end of this range consisting mostly of lymphocytes with few epithelial cells, a middle group consisting of an equal proportion of both epithelial and lymphocytic elements, and, at the opposite end, a tumour containing only scanty lymphocytes in a predominantly epithelial tumour.

In my view the Hodgkin's disease, Grade 1, is a mixed thymic tumour at the lymphocytic end of the scale. The thymic epithelium is represented by the giant cells buried among the lymphocytes and the fibrous stroma of the thymus by the fibrous lobulation of these tumours. The histology is identical to the " paragranuloma " of Jackson and Parker (1947) and " benign Hodgkin's disease " of Harrison (1952). The epithelial giant cells are also identical to the "owl's eyes" already seen in the developing thymus. I agree with Rosenthal (1936) and Lowenhaupt (1948) that the presence of a heavy lymphocytic infiltration in these tumours, being a normal developmental sequence in the thymus, indicates a well differentiated and, therefore, a slowly growing tumour.

I regard the Hodgkin's disease, Grade 2, as a mixed tumour of the thymus which is the pathological " half way house " of the mixed thymic tumour group. The lymphocytes of the thymus are always represented, but in varying degree. The epithelium of the thymus is present in the form of mononuclear cells, " mirror image " giant cells and Dorothy Reed giant cells all of which have their similarities to cells already seen in the developing thymus. These multinuclear cells may, in some examples, form larger cellular masses and approximate to the appearances of a Hassall's corpuscle (Fig. 26). When this occurs the tumour is readily acceptable as thymic in origin. This is also seen in Fig. 20 and 21 which represent an almost pure epithelial thymoma with profuse formation of Hassall's corpuscles. The adjacent epithelial cells of this tumour can be seen to be very similar to many of the cells seen in Hodgkin's disease, although this tumour is clearly of thymic origin and the histological picture, because of the epithelial differentiation and the lack of the other elements, does not simulate the pattern of Hodgkin's disease.

Eosinophil polymorphonuclear leucocytes are a common constituent of the normal thymus, and in Hodgkin's disease these cells are usually present in profusion. Whether they are merely attracted by the presence of additional bulk of thymic epithelium or whether they are an integral part of the tumour remains a matter for conjecture.

The fibrous tissue, which often forms a large part of the neoplastic process, is either a reaction on the part of the tissues to the tumour cells, or is the neoplastic representation of the fibrous lobulation of the normal thymus gland.

Hodgkin's disease, Grade 2, is, therefore, a neoplastic mixture incorporating all the elements of thymic tissue buried in a fibrous stroma of variable density, quantity and cellularity.

The Hodgkin's disease, Grade 3, is a more cellular tumour at the epithelial end of the mixed thymoma scale. The mononuclear tumour cells are epithelial in origin and the giant cells represent abortive and bizarre Hassall's corpuscles. The appearances of these epithelial elements again have their similarities in the developing thymus. The tumour cells evoke less fibrosis, and both the lymphocytes and eosinophils are less numerous. The areas of necrosis are an expression of a rapidly growing tumour, whereby the blood supply is insufficient for the needs of the tumour tissue.

Eisenberg and Sahyoun (1950) describe 7 cases of mixed thymic tumours, all of which had previously been diagnosed as Hodgkin's disease. Only one of these cases was suspected of having a mediastinal primary neoplasm on clinical grounds alone. Chest X-rays showed widening of the superior mediastinum in 5 cases, but the other 2 were radiologically normal. Six of the patients subsequently died following treatment. The extent of spread in these patients shows a remarkable similarity to the 112 cases of Hodgkin's disease already reported in Group I. All had cervical lymph node involvement and a mass in the superior mediastinum. Three showed evidence of pulmonary infiltration, 4 had an enlarged spleen and 2 developed an enlarged liver. Two cases had multiple vertebral deposits and in all there was widespread lymph node invasion. An average prognosis of 53·2 months' survival following the onset of symptoms compared with an average of 37·2 months for cases of Hodgkin's disease previously reported by the same authors (Sahyoun and Eisenberg, 1949).

The interest of Eisenberg and Sahyoun's (1950) publication centres around the striking similarity of the clinical manifestations, the sites of spread and the histological appearances of these thymic cases to those of Hodgkin's disease, in spite of the descriptive and photomicrographic appeals to the contrary.

Lowenhaupt and Brown (1951) published a series of 9 cases of the granulomatous type of thymoma and extracted other examples, considered to represent this type of tumour, from the literature.

At the time of diagnosis all 9 cases had a superior mediastinal mass and 6 had either cervical or axillary node enlargement or chest wall invasion.

Five of the 9 patients subsequently died and the details of the extent of spread of the tumour found at autopsy are described. All showed mediastinal involvement with invasion of either the hilar nodes or the lung. There was cervical lymph node invasion in 5 cases, axillary lymph node and chest wall invasion in 3 cases, with liver invasion in 1 case and splenic involvement in another. The diaphragm was involved in 1 case and the retroperitoneal tissues in 2 cases where the tumour had extended to surround the adrenals, bile ducts, pancreas and posterior aspect of the stomach. Four patients remain alive from $1\frac{1}{2}$ to 5 years later, but 3 have a residual mediastinal mass.

The surviving patients of their series, therefore, have the disease localised to the thymic region with evidence of invasion of the adjacent mediastinal tissues, the cervical or axillary lymph nodes. The fatal cases showed mediastinal, pulmonary and abdominal invasion with either retroperitoneal, splenic or liver involvement. In these fatal cases the extent of spread is therefore analogous to many examples of disseminated Hodgkin's disease and is comparable to the cases in Group I of the present series.

Lowenhaupt and Brown's (1951) paper, with excellent reproductions of chest X-rays, operation specimens and photomicrographs of the histology, deals not only with the extent but also with the method of spread. The authors describe the routes of extension of tumour from the thymic region as anteriorly to involve the sternum, superiorly to the cervical lymph nodes and posteriorly to the tracheobronchial group at the hilum of the lung. These routes of spread follow, therefore, the normal lymphatic paths. There are, in addition, other possible modes of spread from the internal mammary chain to the liver and in the ramifications from the hilar lymph nodes to the thoracic duct to involve the retroperitoneal tissues by retrograde spread.

Both Eisenberg and Sahyoun (1950) and Lowenhaupt and Brown (1951) describe differences between the reticulo-endothelial cells of Hodgkin's disease and the reticulo-epithelial cells of thymic origin. They also note that the thymic giant cells are two to four times larger than the Reed-Sternberg cells of Hodgkin's disease.

In spite of the list of reasons why the mixed cell or granulomatous type of thymoma differs from Hodgkin's disease, I am reluctant to assume that they are, in fact, two separate diseases. The age groups and the clinical manifestations are similar and the sites of dissemination appear identical. A more rational approach is to regard the mixed thymomata and Hodgkin's disease as the same disease process and the variable histological appearances encountered as dependent on the balance between the epithelium and the lymphocytes in an individual tumour, and also on the degree of differentiation of the epithelial cells. With epithelial differentiation more definitive Hassall's corpuscles will be formed and, therefore, the giant cells will be larger. This accounts both for Haagensen's (1932) statement that the giant cells of mediastinal Hodgkin's disease "are different from those of Hodgkin's granuloma," and for the observations of Eisenberg and Sahyoun who describe the thymic giant cells as being larger than those of Hodgkin's disease.

In the present series of 275 cases there were 43 patients with no evidence in their case notes of mediastinal involvement.

Can, therefore, a thymic origin be advanced for all cases of Hodgkin's disease? This is difficult to answer, but a tumour in the thymic region, if small, is notoriously difficult to diagnose. As Jackson and Parker (1947) stress, there is "the necessity for roentgen-ray study of the chest when the patient is first seen, even though there are no symptoms or signs even remotely suggesting such lesions." A lateral chest

X-ray is also essential, as this is the sole method of localising a small tumour in the superior mediastinum, which does not encroach beyond the lateral margins of the sternum (Blalock, 1941). In Group III, many of the patients, having no thoracic symptoms had no chest X-ray and of those that did, many had no lateral radiograph. This certainly is one reason why mediastinal involvement was not detected.

Another possible reason is that this group has a worse average prognosis than Groups I and II, and 16 patients died within a year of diagnosis. It is probable that in these cases the thymic tumour, at the time of death, was not sufficiently large to be detectable clinically. Some of the Group III cases of the present series may, therefore, be similar to many examples of bronchial, mammary and nasopharyngeal tumours which often develop widespread metastases from an occult primary neoplasm.

A further explanation for failure in any type of case to recognise the primary enlargement of the thymic tumour, is that the thymoma may undergo sclerosis due to the fibrous tissue of the tumour. This may occur as part of the natural history of the disease, even before irradiation therapy. Fig. 12 shows a small tumour removed from the thymic region in a case of Hodgkin's disease before any irradiation had been directed at the mediastinum. The illustration shows the prominent fibrosis and the tumour cells are limited to small foci in the dense collagen.

As previously stated, ectopic thymic tissue is present in the neck of a significant proportion of human beings. It is not uncommon to find histologically recognisable thymic tissue removed during block dissections or other surgical procedures in the neck region (Fig. 19).

Gilmour (1937) found ectopic thymic tissue in 21 per cent of dissections of the neck region and Rieffel and Le Mée (1909) in 20 per cent. It is conceivable that tumours of the mixed type may arise from this ectopic thymic tissue and account for the lack of a mediastinal tumour, the primary neoplasm being in the neck.

### CONCLUSIONS.

In my view the embryology of the developing thymus gland gives the clue to the histogenesis of Hodgkin's disease. The tumours of the thymus can be either purely epithelial, purely lymphocytic or a mixture of these. The mixed tumour group is composed, therefore, of both epithelial and lymphocytic elements, and forms the basis of Hodgkin's disease. The mixed tumours show evidence of differentiation along two lines. If there is a profusion of lymphocytes and little epithelium the appearances are those of a Hodgkin's disease, Grade 1. If the epithelium is fully differentiated Hassall's corpuscles are formed in profusion and the tumour is then epithelial, thus outside the range of histological confusion with Hodgkin's disease. A less differentiated epithelial tumour with an admixture of lymphocytes shows both mononuclear cells, giant cells, lymphocytes, eosinophils and fibrous tissue. This is histologically indistinguishable from Hodgkin's disease, Grade 2, the ordinarily accepted picture of Hodgkin's granuloma.

Hodgkin's disease, Grade 3, has an epithelial preponderance, and is a less differentiated example of this mixed tumour with less fibrosis, fewer lymphocytes and eosinophil polymorphonuclear leucocytes. In all these mixed tumours the epithelial cells of both mononuclear and giant cell types have their histological counterparts in the developing foetal thymus.

The failure to find mediastinal involvement in cases of Hodgkin's disease is due to a variety of reasons, but the three probable causes are lack of a chest X-ray, a tumour originating in an ectopic thymus in the neck, or a sclerosis of the primary due to fibrosis.

---

### EXPLANATION OF PLATES.

FIG. 1.—Foetal thymus. Section shows a cross-section of a foetal thymus with a cortex and medulla at 16 weeks. × 12.

FIG. 2.—Foetal thymus. Showing mononuclear vesicular cells in the medulla of the thymus. Note the mitoses. × 800.

FIG. 3.—Foetal thymus. Showing a " mirror image " giant cell in the developing thymus. × 800.

FIG. 4.—Foetal thymus. Showing a " mirror image " giant cell with increased prominence of the nucleoli to give the " owl's eye " appearance. × 800.

FIG. 5.—Foetal thymus. Showing a giant cell with four nuclei centrally arranged. × 800.

FIG. 6.—Foetal thymus. Showing a large multinucleate giant cell. × 800.

FIG. 7.—Foetal thymus. Showing a Hassall's corpuscle. × 800.

FIG. 8.—Foetal thymus. Showing eosinophil polymorphonuclear leucocytes in the developing thymus. × 800.

FIG. 9.—Case 1. Showing the thymectomy specimen. The fibrous lobulation is visible. The longest axis measured 8 cm.

FIG. 10.—Case 2. The chest X-ray shows the thymic mass.

FIG. 11.—Case 3. The chest X-ray shows the thymic mass.

FIG. 12.—Showing a fibrotic mass containing small foci of tumour removed from the thymic site before irradiation. × 1.

FIG. 13.—Case 1. Showing giant cells among lymphocytes. × 100.

FIG. 14.—Case 1. Showing the giant cells surrounded by lymphocytes. This is the histological pattern of Hodgkin's disease, Grade 1. × 500.

FIG 15 —Case 2. Showing a pleomorphic tumour with giant cells and bands of fibrous tissue. × 100.

FIG. 16.—Case 2. Showing the histological picture of Hodgkin's disease, Grade 2, with giant cells, lymphocytes and polymorphonuclear leucocytes. × 500.

FIG. 17.—Case 3. Showing a cellular pleomorphic tumour with conspicuous giant cells. × 100.

FIG. 18.—Case 3. Showing the pleomorphism of the tumour with an " owls' eye " type of giant cell, densely staining giant cells, polymorphonuclear leucocytes and some fibrous tissue. This is the histological pattern of Hodgkin's disease, Grade 3. × 600.

FIG. 19.—Showing ectopic thymus tissue removed from the neck during a thyroidectomy. × 100.

FIG. 20.—Epithelial thymoma. Showing an epithelial thymoma with recognisable but atypical Hassall's corpuscles. × 500.

FIG. 21.—Epithelial thymoma. Showing an epithelial thymoma with a Hassall's corpuscle and mononuclear tumour cells. × 500.

FIG. 22.—Showing " owl's eye " giant cells in a lymphocytic stroma. × 500.

FIG. 23.—Showing " owl's eye " giant cells among lymphocytes and polymorphonuclear leucocytes. × 500.

FIG. 24.—Showing a multinuclear giant cell in a fibrous stroma with lymphocytes and polymorphonuclear leucocytes. × 500.

FIG. 25.—Showing giant cells with prominent nucleoli surrounded by lymphocytes. × 500.

FIG. 26.—Showing a Hassall's corpuscle among lymphocytes. × 500.

Thomson.

9

10

11

12

13 14

15 16

17 18

Thomson.

19

20

21

22

23

24

25

26

Thomson.

The spread of these mixed thymic tumours can be accounted for by a study of the normal lymphatic drainage of the thymus, whereby lymphatic routes extend to the neck, to the chest wall, to the hilar and retroperitoneal lymph nodes.

The reason for the longer survival of patients with thymic tumours restricted to the mediastinum is that they are histologically better differentiated. The well-differentiated epithelial elements lead to a diagnosis of a thymoma of the Hodgkin's type, while if there is even better differentiation, with the formation of diagnostic Hassall's corpuscles, the observer will then call the tumour in question an epithelial thymoma.

In conclusion, therefore, all the essential cellular features of Hodgkin's disease are normally present in the developing thymus. Tumours incorporating a mixture of all these elements, the epithelium with its giant cells, the lymphocytes, the eosinophils and the fibrous tissue—produce the clinical, the histological and the post-mortem features of a syndrome known for over a hundred years as Hodgkin's disease.

### SUMMARY.

It is postulated that Hodgkin's disease has its origin in the thymus gland.

The thymus is composed of epithelial cells, from which are derived the multinucleate Hassall's corpuscles ; there are also lymphocytes, eosinophils and fibrous tissue present.

Hodgkin's disease is regarded as a tumour of the thymus gland in which all these cellular elements, are incorporated in varying degrees to give the histological appearances of Hodgkin's disease.

Histological and pathological evidence, from a series of 275 cases of Hodgkin's disease, is put forward in support of this concept.

The tumours reproduce the appearance of many of the cells seen in the normal developing thymus and an appreciation of the normal lymphatic drainage of the thymus accounts for the sites of spread of Hodgkin's disease.

Where no mediastinal involvement is demonstrable some of the possible reasons are discussed.

I gratefully acknowledge the advice, helpful criticism and assistance I have received from Professor R. W. Scarff and Dr. A. C. Thackray in the course of this investigation. I wish to thank Professor B. W. Windeyer, Mr. T. Holmes Sellors and the Medical and Surgical Staff of the Middlesex Hospital for permission to extract clinical details from the cases under their care. I am indebted to Professor J. D. Boyd, Professor E. W. Walls and Dr. Peter Silver for much anatomical information and embryological material. I am grateful to Dr. A. M. Jelliffe for abstracting much of the clinical material and to Dr. Robin Beare for many translations from the German literature. The expense of this investigation was defrayed by the British Empire Cancer Campaign.

### REFERENCES.

ANDRUS, W. DE W. AND FOOT, N. C.—(1937) *J. thorac. Surg.*, **6**, 648.
BENDA, C.—(1904) *Verh. dtsch. path. Ges.*, **7**, 123.
BILLROTH, T.—(1871) *Wein med. Wehnschr.*, **21**, 1065.
BLALOCK, A.—(1941) *Amer. J. Surg.*, **54**, 149.

BOYD, J. D.—(1950) *Ann. Roy. Coll. Surg.*, **7**, 455.
DECKER, H. R.—(1935) *J. thorac. Surg.*, **4**, 445.
EISENBERG, S. J. AND SAHYOUN, P. F.—(1950) *Arch. Path.*, **49**, 404.
EWING, J.—(1916) *Surg. Gynec. Obstet.*, **22**, 461.—(1940) 'Neoplastic Diseases.' 4th Edition. Philadelphia and London (W. B. Saunders Co.).
GILMOUR, J. R.—(1937) *J. Path. Bact.*, **45**, 507.
GOLDMANN, E. E.—(1892) *Zbl. allg. Path. path. Anat.*, **3**, 665.
GRANDHOMME, F.—(1900) 'Ueber Tumoren des vorderen Mediastinums und ihre Beziehungen zu der Thymudrüse.' Darmstadt (Wittich).
GREENFIELD, W. S.—(1878) *Trans. path. Soc. Lond.*, **29**, 272.
GROSZ, S.—(1906) *Beitr. path. Anat.*, **39**, 405.
HAAGENSEN, C. D.—(1932) *Amer. J. Cancer*, **16**, 723.
HARRISON, C. V.—(1952) *J. Path. Bact.*, **64**, 513.
HEUER, G. J. AND ANDRUS, W. DE W.—(1940) *Amer. J. Surg.*, **50**, 146.
HODGKIN, T.—(1832) *Med.-chir. Trans.*, **17**, 68.
JACKSON, H. AND PARKER, F.—(1947) 'Hodgkin's Disease and Allied Disorders.' New York (Oxford University Press).
JELLIFFE, A. M. AND THOMSON, A. D.—(1955) *Brit. J. Cancer*, **9**, 21.
LOWENHAUPT, E.—(1948) *Cancer*, **1**, 547.
*Idem* AND BROWN, R.—(1951) *Ibid.*, **4**, 1193.
MALLORY, F. B.—(1914) 'Principles of Pathologic Histology.' Philadelphia and London (W. B. Saunders Co.).
MEDLAR, E. M.—(1931) *Amer. J. Path.*, **7**, 499.
PINEY, A.—(1926) *Arch. Path.*, **2**, 301.
PULLINGER, B. D.—(1932) 'Rose Research on Lymphadenoma.' Bristol (John Wright and Sons Ltd.).
REED, D. M.—(1902) *Johns Hopk. Hosp. Rep.*, **10**, 133.
RIEFFEL, H. AND LE MÉE, J.—(1909) *C. R. Acad. Sci., Paris*, **148**, 105.
ROBB-SMITH, A. H. T.—(1938) *J. Path. Bact.*, **47**, 457.
ROSENTHAL, S. R.—(1936) *Arch. Path.*, **21**, 628.
ROUVIÈRE, H.—(1932) 'Anatome des lymphatiques de L'Homme.' Paris (Mason).
SAHYOUN, P. F. AND EISENBERG, S. J.—(1949) *Amer. J. Roentgenol.*, **61**, 369.
STERNBERG, C.—(1898) *Z. Heilk.*, **19**, 21.—(1936) *Ergebn. allg. Path. path. Anat.*, **30**, 1.
STEWART, M. J. AND DOBSON, J. F.—(1924) *Brit. J. exp. Path.*, **5**, 65.
SYMMERS, D.—(1911) *N.Y. med. J.*, **93**, 971.—(1933) *Ann. Surg.*, **95**, 544.
TSUNODA, T.—(1911) *Virchows Arch.*, **204**, 265.
TWORT, C. C.—(1930) *J. Path. Bact.*, **33**, 539.
WALLHAUSER, A.—(1933) *Arch. Path.*, **16**, 522 and 672.
WELCH, J. E.—(1910) *Proc. N.Y. path Soc.*, **10**, 161.
WILKS, S.—(1865) *Guy's Hosp. Rep.*, **11**, 56.
WILSON, F. N. AND PRITCHARD, J. E.—(1945) *Canad. med. Ass. J.*, **53**, 444.
WUNDERLICH, C. A.—(1858) *Arch. physiol. Heilk.*, **17**, 123.